# 

**Published:** 1863-09

**Authors:** 


### CONTENTS.

### ORIGINAL CONTRIBUTIONS.

ART. XXII.—Improved Methods of Treatment in Joint and Spinal Diseases. By E. Andrews, Prof. of Surgery in the Chicago Medical College, ........ 417

ART. XXIII.—Injuries of the Head. By George K. Amerman, Attending-Surgeon to the Chicago City Hospital, ........ 434

ART. XXIV.—Gelsemium in Convulsions. By Dr. Henry Wing, Professor of General Pathology and Public Hygiene in the Chicago Medical College, ........ 438

### PROCEEDINGS OF SOCIETIES.

Meeting of Chicago Medical Society, ........ 444

### SELECTIONS.

Stimulants in the Army, ........ 448

Lecture on Eczema, Including its Impetiginous, Lichenous, and Pruriginous Varieties. By T. McCall Anderson, M.D., ........ 451

On the Prevention of Pitting in Small-Pox, ........ 466

On Rennet Wine, by Dr. George Ellis, ........ 468

Is Alcohol Food? By the Editor of the British Medical Journal, ........ 470

### EDITORIAL.

Alcoholic Stimulants as Prophylactics, ........ 472

Surgeon-General Hammond, ........ 473

Resignation of Prof. H. H. Childs, ........ 474

London Lancet, ........ 474

London Medical Times and Gazette, ........ 474

Braithwaite's Retrospect, ........ 474

The Mechanical Treatment of Angular Curvature of the Spine, ........ 474

Permanganate of Potash as a Disenfectant, ........ 474

Liquor Bismuthi, ........ 475

The Medical and Surgical Journal of the War, ........ 476

Action of Solar Rays on Exposed Intestines, ........ 476

